# Possible positive effect of the *APOE* ε2 allele on cognition in early to mid-adult life

**DOI:** 10.1016/j.nlm.2017.10.008

**Published:** 2017-12

**Authors:** Lindsey I. Sinclair, Christopher W. Pleydell-Pearce, Ian N.M. Day

**Affiliations:** a School of Social and Community Medicine, University of Bristol, Oakfield House, Bristol BS8 2BN, UK; b School of Experimental Psychology, University of Bristol, The Priory Road Complex, Priory Road, Bristol BS8 1TU, UK

**Keywords:** Apolipoprotein E, Memory episodic, Executive functioning, ALSPAC, Genetics, Cognition

## Abstract

**Background:**

ε4 allele possession is associated with an increased risk of Alzheimer’s disease. Its effects earlier in life are less well understood. Previous studies have reported both detrimental effects and a lack of effect on cognition outside dementia. We used genotype based recall from the ALSPAC study to investigate whether *APOE* genotype influences cognition in earlier adult life.

**Methods:**

We invited all individuals with the rarer ε22 or ε44 genotypes and equal numbers of those with ε32, ε33 or ε34 *APOE* genotypes (total n invited = 1936, ages 23–67). Participants were screened for dementia using the Addenbrooke’s Cognitive Examination Revised (ACE-R). Participants were asked to complete a 3 h battery of neuropsychological tests covering a range of cognitive domains. The primary outcome was performance on the Rey Auditory Verbal Learning Test (RAVLT). Transformation of variables was used where required to permit parametric testing. As genotypes are unlikely to be confounded unadjusted analyses were performed.

**Results:**

114 participants were recruited to the study (39 ε33, 27 ε34, 15 ε44, 26 ε32 & 7 ε22). ε4+ participants had higher scores on the cognitive failures questionnaire (10 point increase, p = 0.006) but no deficits on objective cognitive testing. ε2 carriers had slightly better episodic memory performance (p = 0.016), slightly improved n-back accuracy and better executive functioning (trails A&B, p = 0.005).

**Conclusions:**

It is intriguing that the ε2+ group performed better as this group have a lower risk of Alzheimer’s disease. Most previous studies have analysed as ε4/non ε4 so may have missed this effect.

## Highlights

• E4 carriers self reported more memory problems, but no objective differences found.
• E2 carriers performed slightly better on episodic memory test.
• E2 carriers were faster in a test of executive function.

## 1 Introduction

The human *APOE* gene has three alleles: ε2, ε3 and ε4. Possession of an ε4 allele (compared to ε3) has been linked to a higher risk of developing and earlier age at onset of late onset Alzheimer’s disease (AD) (Corder et al., 1993) with evidence of an allele dose effect (Bertram, McQueen, Mullin, Blacker, & Tanzi, 2007). One ε4 allele confers a threefold increase in risk and possession of two ε4 alleles confers an over tenfold increase in risk (Bertram et al., 2007). Possession of an ε2 allele has been linked with a lower risk of AD and may also slow progression of vascular cognitive impairment (Blacker et al., 2007, Corder et al., 1994, Kim et al., 2017, Talbot et al., 1994).

Possession of an ε4 allele is neither necessary nor sufficient for the development of AD and many ε4 allele carriers live to advanced ages with no evidence of dementia (Bunce, Fratiglioni, Small, Winblad, & Bäckman, 2004). It has been suggested that ε4 reduces the age at onset of AD but does not influence whether someone develops it (Meyer et al., 1998). Studies over several decades have reported that ε4 allele possession may have more of a deleterious effect in women (reviewed in (Ungar, Altmann, & Greicius, 2014)).

It has been shown in post-mortem brain studies and in PET studies of older adults that ε4 allele possession is associated with higher levels of amyloid plaques (Caselli et al., 2010, Wirth et al., 2014). In one study this effect was seen even in middle-aged individuals (Ghebremedhin, Schultz, Braak, & Braak, 1998). ε4 effects on neurofibrillary tangles are much less consistent (Raber, Huang, & Ashford, 2004). In contrast individuals with the ε2 variant have less AD neuropathology before extreme old age (Berlau et al., 2009, Ohm et al., 1999).

Most previous studies of the effect of ε4 on cognition in non-demented adults have included older participants. Relatively few have studied younger people (only 8 studies with a mean age of under 50 y in a 2017 meta-analysis) or reported the presence of the ε2 allele (Lancaster, Tabet, & Rusted, 2017). Indeed in one study possession of ε2 was an exclusion criterion (Evans et al., 2014). A meta-analysis in 2011 found evidence of improved episodic memory in ε2 carriers from 6 studies (mean effect size 0.09, 95% CI −0.05 to 0.22) (Wisdom, Callahan, & Hawkins, 2011). The same meta-analysis reported that the adverse effects of ε4 on cognition (in the absence of dementia) increases with age, yet many previous studies have not adequately screened for dementia (Wisdom et al., 2011). In particular many studies have used the mini mental state examination (MMSE) which is much less sensitive to the presence of early dementia (or mild cognitive impairment) than other tests such as the Addenbrooke’s cognitive examination revised (ACE-R) (Mioshi, Dawson, Mitchell, Arnold, & Hodges, 2006).

Although compromised by lack of phenotypic precision, a GWAS meta-analysis which analysed a general cognitive ability score from heterogeneous individual studies found that the effect of ε4 was minimal in middle age and increased with age (Davies et al., 2015). Other studies have reported that the separation between ɛ4 carriers and non-ɛ4 carriers on cognitive tests starts in the mid 50 s (Caselli et al., 2009) and as early as 35 years old (Bunce, Anstey, Burn, Christensen, & Easteal, 2011). The largest recent study of middle aged adults, which used data from the Generation Scotland study, showed detrimental effects of ε4 on logical memory and processing speed. These effects appeared to be larger in those aged >60 y in a sensitivity analysis (Marioni et al., 2016).

There remains considerable debate as to whether the changes seen in some previous studies reflect genuine ε4 effects in the absence of dementia, or merely the early stages of a dementing process. The latter is entirely possible as amyloid can be found in the brain at least 10 years before the diagnosis of a dementia (Fouquet et al., 2014, Morris et al., 2010). Evidence of cognitive decline has been shown 10 and 12 years before the onset of AD (Amieva et al., 2008, Tierney et al., 2005).

Perhaps unsurprisingly, given the known relationship of ε4 to AD risk, most previous studies have included a measure of episodic memory. The cognitive domains of attention, executive function and visuospatial function have been much less well studied (Wisdom et al., 2011). In the 2011 meta-analysis by Wisdom et al. the mean effect size for ε44 homozygotes on episodic memory was −0.18 (95% CI −0.34 to −0.02) and for ε34 participants it was −0.04 (95%CI −0.09 to 0.01). There was also evidence of small ε4 effects on executive functioning (effect size −0.06, 95% CI −0.12 to −0.04) and perceptual speed (effect size −0.07, 95% CI −0.13 to −0.01) (Wisdom et al., 2011). Although episodic memory is the major cognitive process affected early in LOAD, it is not the only process affected early in the disease process (Backman, Jones, Berger, Laukka, & Small, 2005). Poor working memory performance has been reported as one of the earliest deficits seen in Alzheimer's disease (McKhann et al., 1984). The current study builds on previous work by including younger participants, including a relatively large number of ε2+ participants, rigorously screening for dementia (to exclude this as a cause of any differences observed) and by testing multiple domains of cognition.

Whilst some individual studies have suggested that there is a positive pleiotropic effect of ε4 in younger adults (e.g. Hubacek et al., 2001) a meta-analysis found no evidence of such an effect (Ihle, Bunce, & Kliegel, 2012). The authors suggested that this may have been because several of the studies used less difficult tasks that may have failed to pick up subtle effects.

We wished to study the effects of *APOE* genotype on cognition independent of dementia. We hypothesised that cognitive function is reduced in young to middle aged adults without dementia from the ALSPAC study with an ε4 allele compared to those without.

Recall by genotype is an efficient study design which allows causal inference, precision phenotyping and maximises statistical power (Ware, Timpson, Davey Smith, & Munafò, 2014). It involves selecting a defined number of participants with each genotype at random from an existing study with genetic data. These participants are then invited to take part in the recall study. Dense phenotyping can be performed in the recall study which would be impracticable to perform on the whole study cohort.

## 2 Methods and materials

### 2.1 Ethics

Ethical approval for this study and approval for substantial amendments was provided by the ALSPAC ethics and law committee (study ref E201109). Both researchers and participants were blind to *APOE* genotype status. All participants gave written informed consent to take part in this study following assessment of their capacity to do so by a Psychiatrist (LIS).

### 2.2 The Avon longitudinal study of parents and children

The Avon Longitudinal Study of Parents and Children (ALSPAC) as described previously in detail is a prospective study which was established in 1991 (Boyd et al., 2012, Fraser et al., 2012). Initially 14,541 women were enrolled, resulting in 14,062 live births and 13,988 children alive at one year. At age 7/8 further eligible children were added to the sample, giving a total sample size of 15,247 eligible pregnancies and 14,775 live births. Data were collected from self-report questionnaires, teacher report questionnaires, medical, educational and other records, birth registries, and hands on assessment. Detailed information has been collected since birth via questionnaires and at regular clinics. The study website contains details of all the available data through a fully searchable data dictionary (http://www.bris.ac.uk/alspac/researchers/data-access/data-dictionary/).

### 2.3 APOE genotyping

Genotyping of all study participants for *APOE* was undertaken by integrated single label liquid phase assay as previously described (Abdollahi et al., 2006). DNA samples were available in 2009 for 7091 children, 63% of the 11343 ALSPAC children with potential DNA samples available. In total 95% of these samples were genotyped (Taylor et al., 2011). After siblings and children of known non-white ethnicity were excluded there was genotype data for 5995 children. DNA samples were available in 2010 for 9763 mothers: 83.6% of the 11,679 mothers with potential DNA samples available. In total 87.9% of these samples were able to be genotyped. There was no strong evidence of a sex difference in genotype distribution or of a deviation from Hardy Weinberg Equilibrium for either the mothers or young people (p > 0.05).

### 2.4 Recall by genotype

Inclusion criteria for this study were that participants had previously taken part in the ALSPAC study and that they had a known *APOE* genotype. Exclusion criteria were; drop out from ALSPAC; living >50 miles away; already taking part in an ALSPAC sub-study; very poor command of English; lack of capacity to consent; and dementia.

**Fig. 1:**
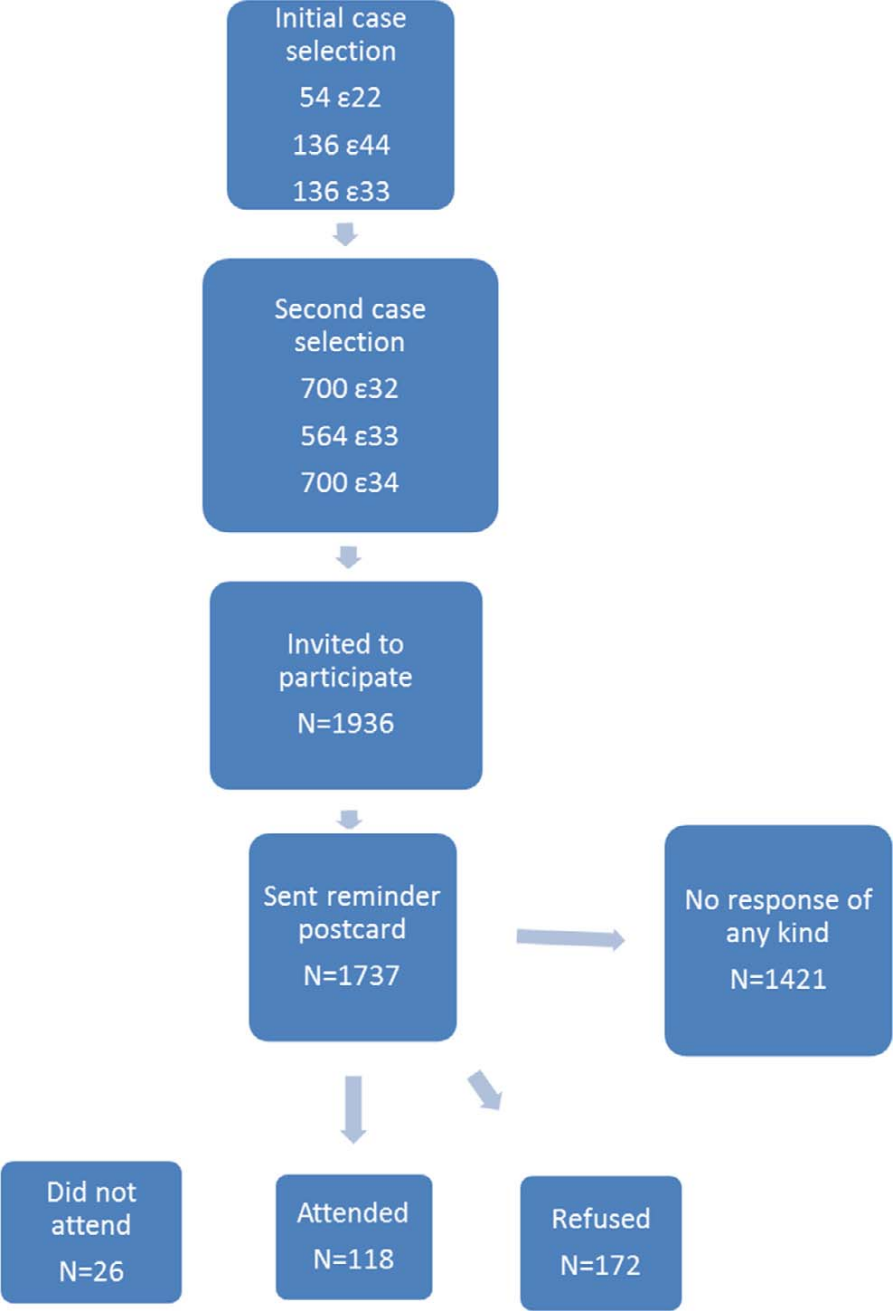
The flow of potential participants through the study.

Recruitment to the study is summarised in Fig. 1. The planned strategy was to identify whether a per-genotype difference existed between the homozygotes and then to ascertain whether there was an allele dose effect by inviting heterozygotes in a second wave. Due to a very poor response rate to the first wave the invitations to the second case selection were sent earlier than planned.

Participants in each batch of invitations were chosen at random from the case selection. They were sent an initial invitation, a reminder postcard after 3 weeks (if they had not replied) and if they had still not replied were contacted by telephone. All participants were reminded the day prior to their appointment. In order to maintain double blinding as to participant genotype, all invitation letters, case selections, telephone calls and participant contact other than the study visit were carried out by ALSPAC staff and not by the researchers.

Despite all of these measures the final response rate (either positive or negative) was only 16%. Participants and the rest of the ALSPAC study population were compared (see Supplementary Table 1).

### 2.5 Co-variates

Information was available on a range of possible confounders including IQ, blood pressure, serum cholesterol, past medical history, mood rating scales and demographic variables.

### 2.6 The study visit

The study visit lasted for up to 3 h. Participants were screened for dementia using the ACE-R, which incorporates the MMSE (Mioshi et al., 2006). Information was gathered on head injuries, history of mental illness, current medication, caffeine consumption, alcohol intake, illicit drug use and family history of dementia. All young people completed the moods and feelings questionnaire (Angold et al., 1995). All mothers completed the Edinburgh Postnatal Depression Scale (EPDS) (Cox, Holden, & Sagovsky, 1987) and the Crown-Crisp Inventory (Crown & Crisp, 1966). These questionnaires were chosen to allow comparison with data previously collected by ALSPAC. All participants completed the Depression, anxiety and stress scale (DASS) and the Cognitive Failures Questionnaire (CFQ) (Broadbent et al., 1982, Lovibond and Lovibond, 1995a, Lovibond and Lovibond, 1995b). The DASS may be downloaded from www.psy.unsw.edu.au/dass/. Full scale IQ was measured using the Wechsler abbreviated scale of intelligence (WASI) (Wechsler, 2011).

The neuropsychological battery included the Rey Auditory Verbal Learning Test (RAVLT), bespoke episodic list learning and paired associative list learning tasks, a Stroop test, digit span, n-back, verbal fluency (FAS and animals), the Rey Osterrieth complex figure, trails A&B, simple & choice reaction time and a bespoke visual motion task (Borkowski et al., 1967, Kirchner, 1958, Rey, 1941, Rey, 1964, Rm, 1955, Spreen and Strauss, 1998, Stroop, 1935, War Department Adjutant General’s Office, 1944, Wechsler, 1997) Tests were carried out in a standard order and breaks were offered at two regular points. No feedback was offered to participants on how they had performed on the tests. The researcher was present during all tests. Participants were requested to refrain from smoking and to drink only one cup of their normal caffeinated beverage the morning of the study visit. This was to avoid the known effects of caffeine on performance in such tests (Ker, Edwards, Felix, Blackhall, & Roberts, 2010).

Participants were read standardised instructions for each task immediately before completing them. In the simple reaction time task participants were asked to press a response key as soon as they saw the stimulus. The interval between trials was a random value between 1000 ms and 1500 ms. In the choice reaction task participants were asked to press one key if the stimulus was red and a different key if it was green. The interval between trials was a random value between 1000 ms and 2000 ms.

In the Stroop task participants were requested to push one of three keys each time a stimulus was displayed, to indicate if the colour of the word was red, green or blue. Congruent, incongruent and neutral stimuli (XXX of the same length as the other words) were displayed until the participant responded. The interval between trials was a random value between 1000 ms and 1500 ms. There were up to 36 trials of each condition.

For the n-back task participants were asked to press the space bar if they recognised a letter as being the same as n letters back. The response key was the same in all conditions. Each letter was displayed for 2 s with a 1 s interval between letters.

During the visual motion task participants were asked to push; one key if the stimulus was going in the same direction as the other objects; and a different key if the stimulus was going in a different direction to the other objects. There were 3 different speeds that the stimulus moved at, with 30 trials at each speed. Each trial moved to the next when the participant pressed a key.

### 2.7 Statistical analysis

All analyses were carried out in STATA v13. There was no evidence to support an association of any demographic variables or potential confounders with *APOE* genotype (Table 1). Given that participants with each genotype were selected at random and that genotypes are unlikely to be confounded in any case, it was decided not to include any co-variates in the analysis.

**Table 1:** Characteristics of the study population.

Variable	ε2+		ε33		ε4+		Statistical evidence (ANOVA or Mann Whitney)
	Mean	SD	Mean	SD	Mean	SD	
Within case selection							
Took part in study	33		39		42		p⩽0.0001^*^
Did not take part in study	721		661		788		
Mean age of mothers at visit (yrs)	51.424	4.867	50.282	3.993	51.143	4.459	p = 0.513
*Gender*							
Male	20		15		20		Χ^2^ = 3.521
Female	13		24		22		p = 0.172
Young Person (YP)	22		21		23		Χ^2^ = 1.473
Mother	11		18		19		p = 0.479
IQ	114.333	7.292	112.921	11.741	113.488	10.107	Χ^2^ = 0.208
							p = 0.901
Mini Mental State Examination (MMSE) score	29.788	0.415	29.553	0.86	29.667	0.612	Χ^2^ = 0.732
							p = 0.693
Addenbrooke’s Cognitive Examination (ACE-R) score	93.909	3.076	93.676	4.137	93.167	3.963	Χ^2^ = 0.460
							p = 0.795
Positive family history of dementia (%)	0.394		0.297		0.415		Χ^2^ = 1.488
							p= 0.475
History of a significant head injury (%)	0.152		0.263		0.195		Χ^2^ = 0.217
							p = 0.897
No. of participants who use cannabis regularly	3.500	0.707	0	0	0.750	1.5	N/A
No. of participants who use stimulants regularly	0.500	0.707	0	0	0	0	N/A
No. of cups of caffeine containing drink usually consumes per day	2.604	2.04	3.459	2.34	3.839	3.144	Χ^2^ = 3.034
							p= 0.219
No. of cups of caffeine containing drink consumed that day	0.613	0.715	0.73	0.871	0.902	1.934	Χ^2^ = 0.046
							p= 0.977
Units of alcohol per week	10.348	12.519	9.456	10.997	7.488	8.092	Χ^2^ = 0.708
							p= 0.702
Personal history of epilepsy	0.061		0.026		0.024		Χ^2^ = 0.895
							p = 0.639
History of anxiety disorder	0.121		0.237		0.190		Χ^2^ = 1.445
							p = 0.486
History of depression	0.121		0.184		0.262		Χ^2^ = 2.418
							p= 0.298
History of psychotic disorder	0		0		0		N/A
Crown-Crisp total score	25.697	36.987	35.526	38.2	32.667	36.701	Χ^2^ = 3.006
							p= 0.222
Cognitive Failures Questionnaire Score	30.121	13.944	32.263	11.95	38.929	15.371	Χ^2^ = 6.051
							p = 0.049^*^
Last available cholesterol (mothers) mmol/L	4.813	0.9	5.202	0.913	5.049	0.536	p < 0.001^*^
Depression, Anxiety and Stress Scale (DASS) total score	9.424	9.572	7.553	6.769	17.214	16.389	Χ^2^ = 7.487
							p = 0.024^*^
Edinburgh Postnatal Depression Scale (EPDS) total score	4.212	6.143	5.789	6.338	5.69	6.426	Χ^2^ = 1.022
							p = 0.600
Moods & Feelings total score	3.061	2.85	2.421	2.937	3.524	4.49	Χ^2^ = 0.375
							p = 0.829

A power calculation estimated a total n of 350 i.e. 70 in each genotype group to ensure 80% power with an α of 0.05. It soon became apparent that this was not going to be possible so numbers were maximised wherever possible. To maximise study power an analysis strategy of combining ε32 and ε22 to form an ε2+ group and combining ε34 and ε44 to form an ε4+ group was used with ε33 as the reference group.

Transformation of variables was used where necessary to permit parametric testing. Where parametric testing was possible the data was initially analysed using ANOVA and subsequently by linear regression. Non-parametric testing was performed using the Kruskal-Wallis and Dunn’s post hoc tests. Effect sizes were calculated, where possible, using the post hoc estate size command in Stata after ANOVAs.

For the N-back data multi-level regression was used, as described previously (Sinclair, Button, Munafò, Day, & Lewis, 2015). Unfortunately reaction times were not available for target/non-target so the reaction time data was only re-shaped according to the difficulty level. It was not possible to use multilevel regression for accuracy in the visual motion task as the data had a truncated normal distribution, but it was possible to use a repeated measures ANOVA.

For the simple reaction time all unfeasibly long (>1000 ms) or short (<120 ms) reaction times were excluded before calculation of mean and median reaction times. A further corrected (C-mean) mean reaction time was calculated by excluding all reaction times >2SD away from the sub-mean. The C-mean was then used in analyses. For the choice reaction time task the same procedure was followed, except that the upper limit for excluding unfeasibly long reaction times was raised to 2000 ms. The difference between the choice C-mean and simple C-mean was calculated and used in the analysis as this is a purer measure of choice than the choice reaction time alone.

We made an a priori decision to exclude individuals with an ε42 genotype from analyses as this group mixes the high and lowest risk alleles. This is standard practice in *APOE* research (e.g. Alexander et al., 2007) We also made an a priori decision to exclude those who scored <82 on the ACE-R as this has optimal sensitivity for dementia (Mioshi et al., 2006).

## 3 Results

### 3.1 Demographics

The study population differed by *APOE* genotype from those in the case selection who did not take part (see Table 1). In particular homozygotes for rare alleles were more likely to participate. The cause for this is unknown. Although homozygotes were invited first and then heterozygotes in the second wave of invitations the case selection was performed at random (other than for homozygotes for rare genotypes who were all invited to participate) and participants were then invited in a random order. There was no difference in the rate of family history of dementia between the groups. The expected effect of *APOE* genotype on serum LDL cholesterol was observed (Zacho et al., 2008). The age range of those who took part was 23–24 (young people) and 42–67 (mothers). There were 33 individuals with an ε2+ genotype with an age range of 23–24 (young people) and 43–67 (mothers). There were 39 individuals with an ε33 genotype whose ages ranged from 23 to 24 (young people) and 42 to 62 (mothers). Finally there were 42 individuals with an ε4+ genotype, with ages ranging from 23 to 24 (young people) and 42 to 62 (mothers).

### 3.2 Test battery results

There was little evidence of a per genotype difference in performance on the reaction time, digits forwards/backwards task, the Rey-Oesterrieth complex figure task or the Stroop test (see Table 2). Although the initial ANOVA was suggestive of a per genotype difference in phonemic fluency (p = 0.047) no evidence of a difference was seen in a subsequent linear regression. There was no evidence of a per genotype difference in category fluency (p = 0.647).

**Table 2:** Results from the neuropsychological test battery. Χ^2^ refers to statistical evidence from the Kruskal Wallis test.

Variable	ε2+		ε33		ε4+		Statistical evidence (ANOVA or Kruskal Wallis)
	Mean	SD	Mean	SD	Mean	SD	
Digits forwards	6.061	1.029	5.925	1.185	5.907	1.25	p = 0.835
							Effect size = 0.003
Digits backwards	4.576	1.437	4.3	1.381	4.186	1.367	p = 0.496
							Effect size = 0.013
RAVLT trials I-V recall (errors)	1.636	2.133	1.425	2.024	1.419	1.918	Χ^2^ = 1.205
							p = 0.547
RAVLT trials I-V recall (repetitions)	15.424	12.281	9.05	10.583	13.791	15.239	Χ^2^ = 4.650
							p = 0.098
RAVLT trials I-V recall (total)	57.848	7.484	52.7	10.118	53.884	7.582	Χ^2^ = 6.663
							p = 0.036^*^
RAVLT trial VII delayed recall (errors)	11.969	2.901	10.75	3.095	11.214	2.968	Χ^2^ = 0.523
							p = 0.770
RAVLT trial VII delayed recall (total)	11.969	2.901	10.75	3.095	11.214	2.968	Χ^2^ = 2.980
							p = 0.225
Verbal fluency (FAS) total no. words	43.697	8.432	41.25	11.047	41.186	11.065	p = 0.567
							Effect size = 0.010
Category fluency (animals)	23.636	3.959	21.85	5.323	23.093	4.83	p = 0.355
							Effect size = 0.018
Paired associative learning (errors)	1.667	1.931	1.725	1.961	2.465	2.693	Χ^2^ = 2.907
							p = 0.234
Paired associative learning (total)	51.242	7.08	45.7	13.921	46.651	9.768	Χ^2^ = 4.722
							p = 0.094
Rey-Osterrieth figure delayed recall	22.788	4.697	20.075	7.604	21.093	6.045	p = 0.317
							Effect size = 0.029
Rey-Osterrieth figure immediate recall	23.561	5.275	19.738	8.252	21.244	6.073	p = 0.104
							Effect size = 0.040
Trails B – Trails A (secs)	20.595	9.636	36.745	41.301	33.835	23.521	Χ^2^ = 11.704
							p = 0.003^*^
Episodic list learning total recalled	11.697	2.687	10.075	3.214	10	3.471	Χ^2^ = 6.842
							p = 0.033^*^
Episodic list learning errors	0.424	0.792	0.475	0.816	0.31	0.643	Χ^2^ = 0.781
							p = 0.677
C-mean simple reaction time (ms)	292.049	38.829	284.925	34.884	289.379	29.281	Χ^2^ = 0.839
							p = 0.657
C-mean choice reaction time (ms)	438.109	59.592	440.356	60.409	446.225	53.149	p = 0.778
							Effect size = 0.004
Choice RT error rate	0.036	0.021	0.041	0.029	0.047	0.042	Χ^2^ = 1.990
							p = 0.370
Stroop interference effect (ms)	91.878	95.07	124.454	94.421	109.775	84.805	p = 0.324
							Effect size = 0.020

### 3.3 Episodic memory

**Fig. 2:**
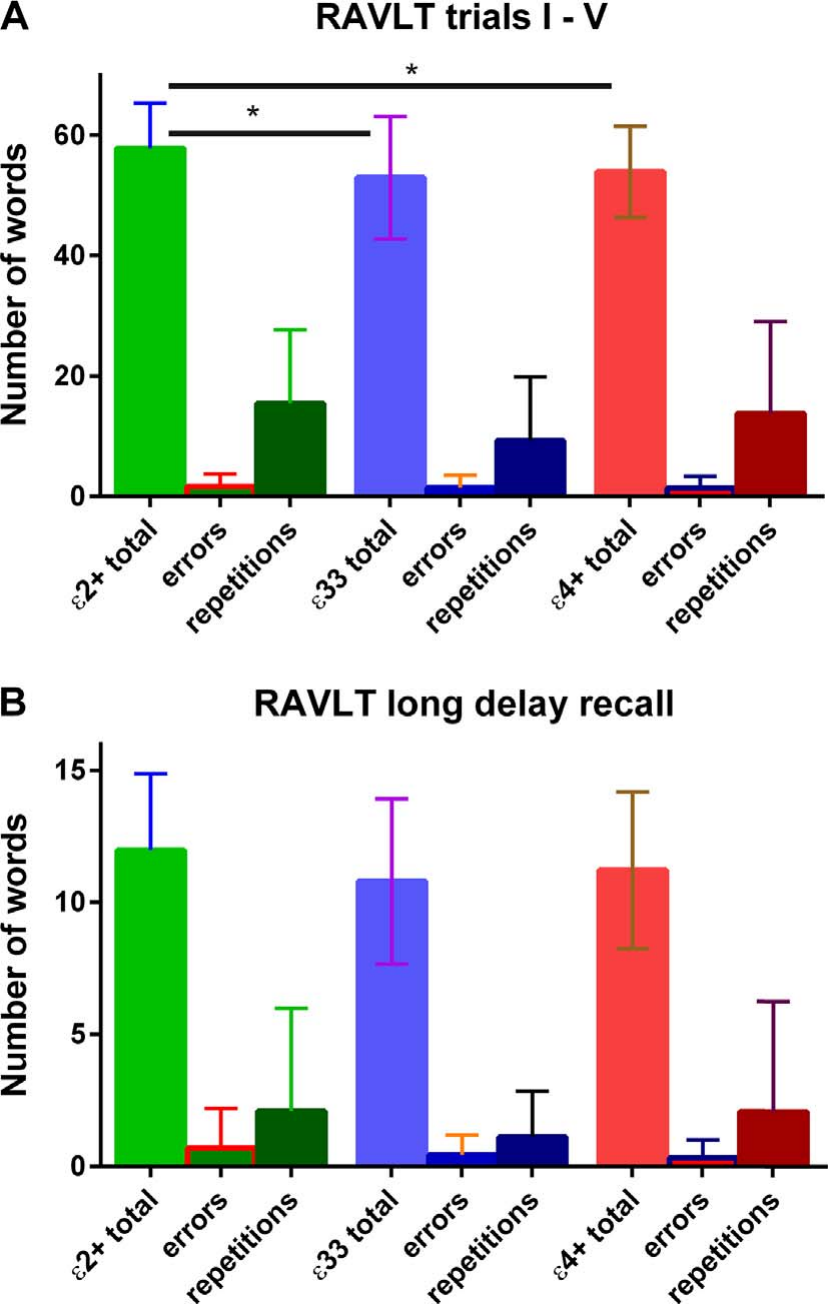
Results from the RAVLT. There was evidence that the ε2+ group remembered more words in trials I-V (A) but no statistical evidence to support a between group difference at the long delay time point (B). The standard deviations for each group are shown in the error bars. A post hoc Dunn’s test suggested that the ε2+ group remembered more words than either the ε33 group (p = 0.011) or the ε4+ group (p = 0.012). ^*^ denotes p < 0.05.

**Fig. 3:**
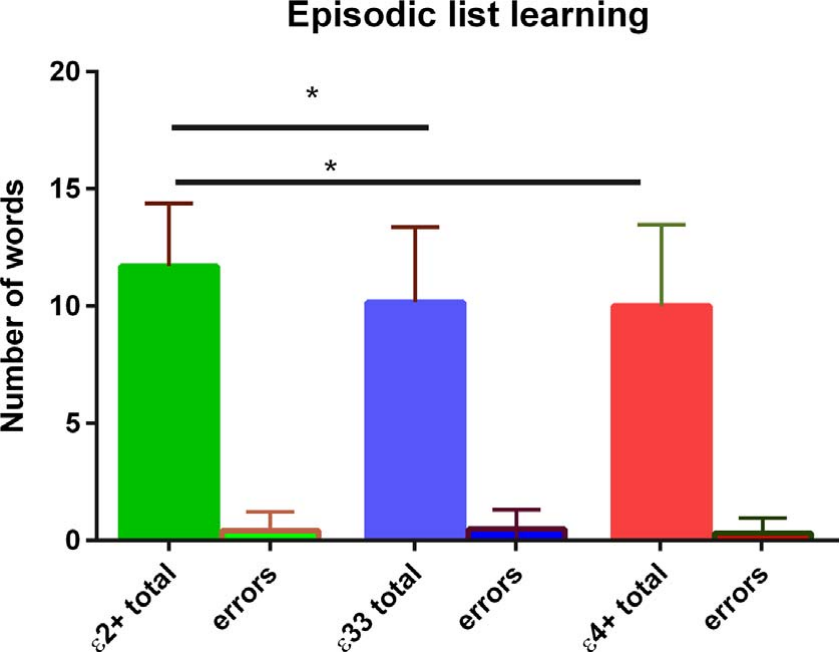
Episodic list learning. The standard deviations for each group are shown in the error bars. There was evidence from a post-hoc Dunn’s test that the ε2+ group performed better than the ε33 (p = 0.016) group and the e4+ group (p = 0.007). ^*^ denotes p < 0.05.

In the main analysis there was no evidence of a per genotype difference in performance at the long delay timepoint in the RAVLT (see Fig. 2B). As shown in Fig. 2A the ε2+ group remembered slightly more words in the first 5 trials of the RAVLT (5.1 more words, Χ^2^ = 6.663, p = 0.036) In addition there was evidence from both the Kruskal-Wallis and Dunn’s tests that ε2 carriers performed better on the episodic list learning task (1.6 more words, Χ^2^ = 6.842, p = 0.016) (see Fig. 3). This was an immediate recall task. There was no evidence of a per-genotype difference in the paired associative learning task.

### 3.4 Executive functioning

**Fig. 4:**
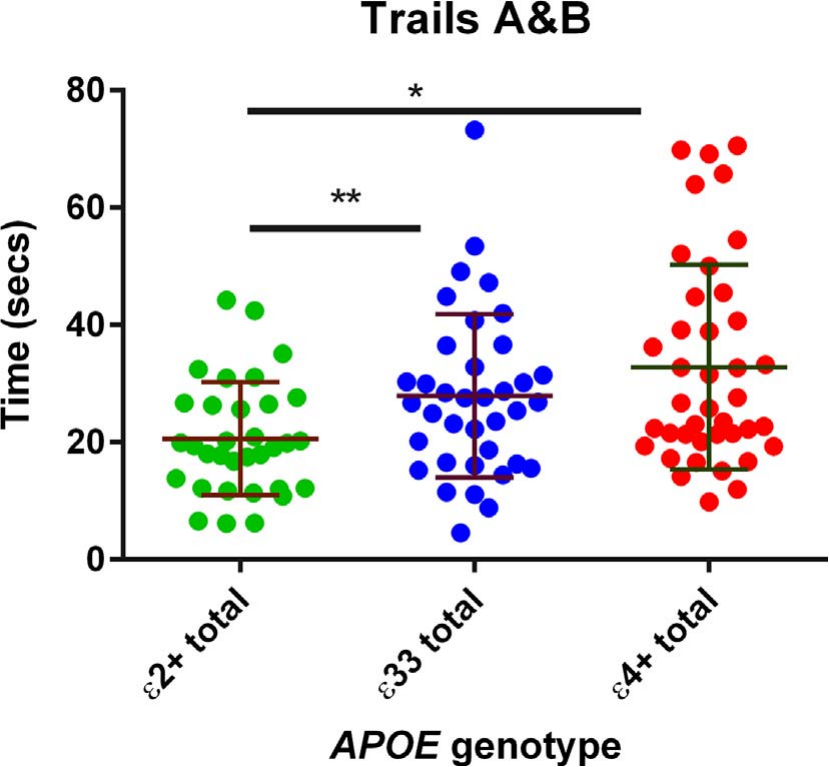
The results shown are the time taken to complete the trails B task minus the time taken to complete the trails A task. Standard deviations for each group are shown in the error bars. There was evidence from a post-hoc Dunn’s test that the ε2+ group performed better than the ε33 (p = 0.005) group and the ε4+ group (p < 0.001). ^*^ denotes p < 0.05 and ^**^ denotes p < 0.01.

There was reasonable strength evidence that those with an ε2 allele were faster (i.e. performed better) in the trails A&B test (Χ^2^ = 11.704, p = 0.003, see Fig. 4). The Kruskal-Wallis and Dunn’s post hoc test suggested that there was a difference between the ε2+ group and the ε33 (p = 0.005) and the ε4+ group (p < 0.001). This should be interpreted with caution.

### 3.5 Working memory

As would be expected the participants had almost perfect accuracy on the 1-back task (see Table 3). Accuracy was lower for the 2-back and much lower for the 3-back, as anticipated. Neither the accuracy variable nor d’ were normally distributed (truncated normal distribution with an upper limit of 1.0) and it was not possible to transform them. It was not possible to calculate d’.

**Table 3:** Results from the n-back task at levels 1, 2 and 3 back.

		ε2+		ε33		ε4+	
Accuracy		Co-efficient	P-value	Co-efficient	P-value	Co-efficient	P-value
	Multi-level regression	0.036 (0.003–0.069)	0.034^*^	Reference group		0.008 (−0.23 to 0.040)	0.590
	LR test for overall effect	p = 0.091					
Reaction time		Co-efficient	P-value	Co-efficient	P-value	Co-efficient	P-value
	Statistical evidence from multi-level regression	−0.001 (−0.111 to 0.110)	0.987	Reference group		−0.021 (−0.125 to 0.083)	0.694
	LR test for overall effect	p = 0.888					

The regression was therefore performed with bootstrapping as this method does not require a normal distribution. The residuals from this regression were normally distributed. The results were unchanged with bootstrapping. There appeared to be improved accuracy (3.6% increase, β = 0.036 (95% CI = 0.003–0.069), p = 0.034) in the ε22 group, although the overall likelihood test for an effect of *APOE* suggested that there was no effect (p = 0.091, see Table 3). The overall r2 was 0.473.

There was no evidence that *APOE* genotype influenced reaction time to target in the 2- or 3-back n-back task (p = 0.888, see Table 3).

### 3.6 Visual motion task

The motion task had 3 levels of speed and 2 levels for target i.e. present/absent. One participant was excluded because they had a large number of unfeasibly fast reaction times (<120 ms) and their accuracy was at chance level. When calculating the reaction time all unfeasibly short (<120 ms) reaction times were excluded before calculation of mean and median reaction times. A further corrected (C-mean) mean reaction time was calculated by excluding all reaction times >2SD away from the sub-mean. Only 1 trial from one participant was excluded because of a reaction time <120 ms.

Multi-level regression of accuracy was not possible because a normal distribution could not be obtained. The analysis proceeded using d’ as this takes account of both accuracy to target and false alarms. The discriminability index (better known as d’) is a measure of signal strength that takes account of signal and “noise”. To prevent a false alarm rate of 0 (and thus an infinitely large d’) where the false alarm rate was zero it was adjusted to be 1/N where N was the number of valid trials for that participant.

Because the within subject co-variance of the d’ data structure was compound symmetric it was possible to use a repeated measures ANOVA. There was no evidence that *APOE* genotype had an effect on d’ (see Table 4 and supplementary Tables 4 and 5).

**Table 4:** C mean reaction times and accuracy rates for the different conditions in the visual motion task. ^*^denotes p < 0.05.

			ε2+		ε33		ε4+	
			Mean	SD	Mean	SD	Mean	SD
Accuracy	Target absent	Fast	0.989	0.02	0.945	0.108	0.974	0.033
		Medium	0.98	0.029	0.961	0.078	0.981	0.029
		Slow	0.983	0.027	0.952	0.097	0.977	0.040
	Target present	Fast	0.954	0.054	0.929	0.100	0.940	0.068
		Medium	0.939	0.055	0.921	0.107	0.930	0.085
		Slow	0.939	0.066	0.943	0.085	0.946	0.091
	Repeated measures ANOVA		*APOE* genotype		p = 0.588			
			Interaction between *APOE* genotype and level		p = 0.027			

C mean reaction time (msec)	Target absent	Fast	1235.67	756.354	1072.34	820.401	1065.691	411.334
		Medium	1351.498	1173.153	1065.149	781.691	1079.731	456.079
		Slow	1371.326	1114.924	1108.585	879.918	1089.68	483.82
	Target present	Fast	819.296	301.045	740.199	324.114	750.257	252.855
		Medium	828.271	316.083	747.454	258.899	783.043	264.319
		Slow	993.369	488.757	847.204	316.613	868.535	306.196
	Statistical evidence from multi-level regression		Co-efficient	P-value	Co-efficient	P-value	Co-efficient	P-value
			−0.014 (−0.30 to 0.002)	0.106	Reference group		−0.007 (−0.023 to 0.008)	0.354

Multilevel regression for reaction time was possible by inverse transformation of reaction time (see Table 4 and supplementary Table 6). There was no evidence of an *APOE* effect on reaction time.

### 3.7 Self-reported cognitive difficulties

**Fig. 5:**
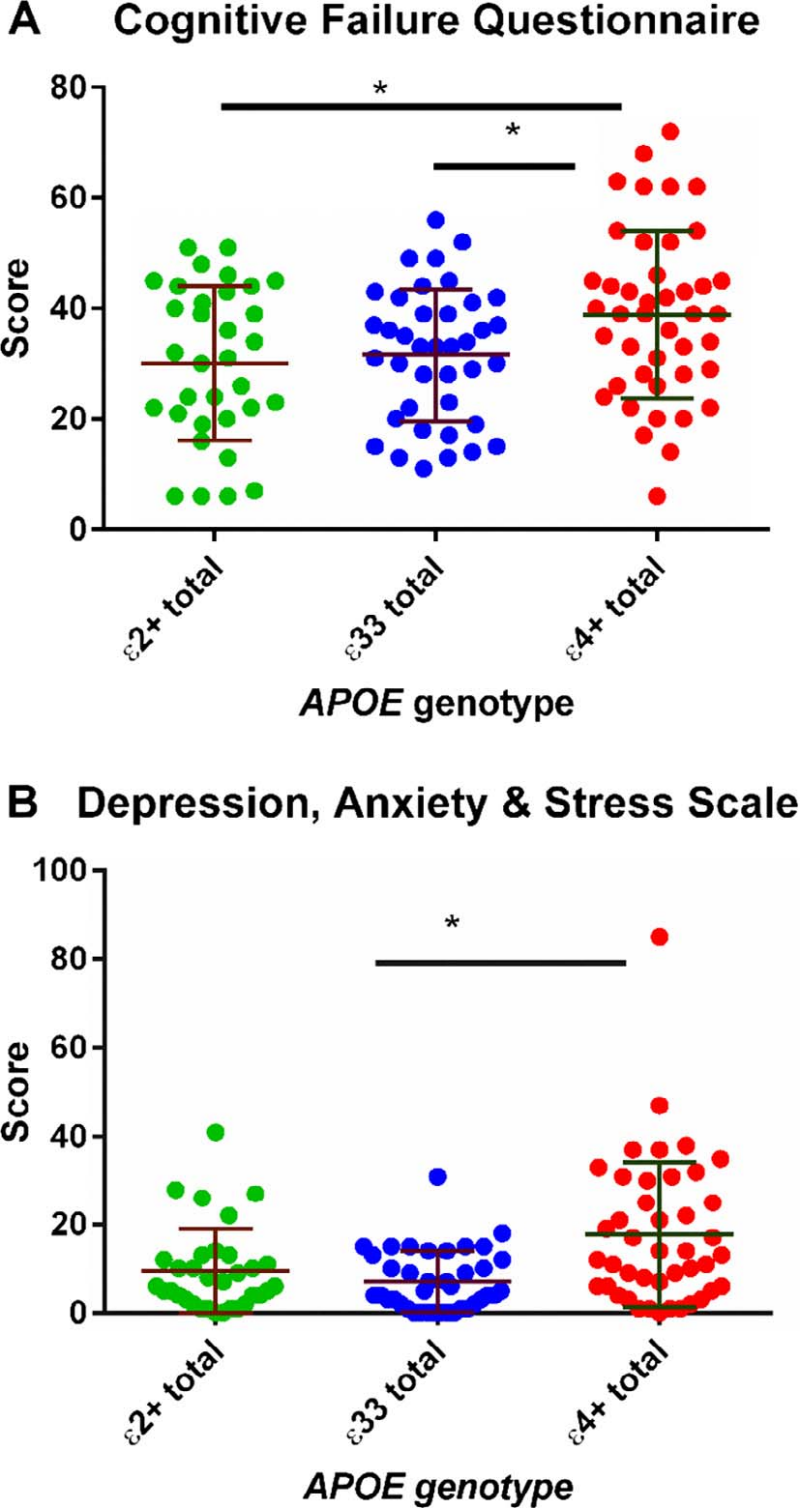
Results from the CFQ (A). Standard deviations for each group are shown in the error bars. There was evidence from a post-hoc Dunn’s test that the ε4+ group had higher scores than the ε33 (p = 0.021) group and the ε2+ group (p = 0.015). As shown in figure B the ε4+ group had higher scores on the DASS. ^*^ denotes p < 0.05.

There was evidence from the cognitive failures questionnaire (CFQ) that self-reported cognition was worse in those with an ε4 allele (Χ^2^ = 6.051, p = 0.006, see Fig. 5A). Those with an ε4 allele also scored somewhat higher on the depression anxiety and stress scale (DASS). The overall Kruskal-Wallis test and the Dunn’s post-hoc test (see Fig. 5B) suggested that those with an ε4 allele scored higher on this scale than either of the other two groups (increase = 9.7 points, overall p value = 0.024, Χ^2^ = 7.487, comparison to ε33 group p = 0.009)

### 3.8 Effect of age group

The dichotomised age groups in this study allowed us to examine differences in observed *APOE* genotype effects between young people and middle aged women. Unfortunately low numbers precluded statistical analysis, but the data are presented graphically in Supplementary Figs. 1–7. It would seem that the ε2+ positive effect on episodic memory was seen predominantly in the young people and the ε2+ effect in the trails A&B test was seen in both age groups. It appears that ε4 had slightly more effect on DASS scores in the mothers, which is surprising as there were no per genotype effects on either the Crown-Crisp or the EPDS which overlap somewhat with the DASS. Finally it seems that the higher scores on the CFQ for individuals with an ε4+ genotype was driven by the young people. Due to low numbers these findings are speculative and impossible to prove.

## 4 Discussion

We found evidence to suggest that ε4 allele possession is associated with more self-reported cognitive failures, but minimal objective difference in cognitive performance compared to the reference ε33 group. We also found that possession of an ε2 allele is associated with slightly better episodic memory performance, slightly improved accuracy on the n-back task and better executive functioning (as measured by trails A&B) than the reference ε33 group. There was no evidence of a per genotype difference in reaction time, attention, verbal fluency or working memory. Effect sizes, where it was possible to calculate them, were very low. It is intriguing that the ε2 carriers, who have an up to 50% reduction in the risk of AD should demonstrate advantage in episodic memory and executive functioning tasks as these are cognitive domains affected early in the development of AD. Although Wisdom et al. found a beneficial effect of ε2 on episodic memory, the very large generation Scotland study (which had ten times the number of ε2 carriers) found no effect of ε2 on any cognitive domain examined (Marioni et al., 2016, Wisdom et al., 2011).

In a 2011 meta-analysis Wisdom et al. found that ε2 carriers performed better than ε4 carriers in episodic memory tests (effect size *d* = 0.09, p < 0.05) but they did not find any statistical evidence of difference from the reference ε33 group. This was based on 6 studies and a total n of 270 ε2 carriers (Wisdom et al., 2011). This finding is consistent with previous research. For example, the Religious Orders study found that episodic memory performance in ε2 carriers did not decrease with age, unlike ε33 and ε4 carriers, although at least 15% of its cohort developed AD during the ≤8 y follow-up period (Wilson, Bienias, Berry-Kravis, Evans, & Bennett, 2002). A similar Finnish community based study with a 3 y follow-up had similar results (Helkala et al., 1996). In a secondary analysis of data from an existing longitudinal study aimed at examining the effect of cardiovascular risk factors Blair et al. demonstrated that individuals with an ε2 allele had the least cognitive decline over a 6 y period (Blair et al., 2005). This was a large study, with mixed ethnicity and a mean participant age of 55. Its main weaknesses were a loss to follow-up and a limited test battery. Conversely the HALCYON study, a combination of ageing cohorts, found no meta-analytic evidence of an ε2 effect on episodic memory or verbal fluency, although this was largely based on cross-sectional measures (Alfred et al., 2014). The majority of participants came from studies with a mean age in the mid 50 s. Given that many previous studies have analysed their results as ε4+ vs. ε4− it is possible that if ε2 does have a beneficial effect on cognition then including them in the ε4− (control) group may have possibly inflated findings that ε4 allele possession has a detrimental effect on cognition.

A meta-analysis in 2011 found a small effect of ε4 on episodic memory in adults without dementia, but gave the caveat that many studies had not adequately screened for dementia. Most of the studies had included mainly older adults. More recent studies have also found an effect of APOE genotype on episodic memory in later adult life, for example the limited GWAS by Andrews et al. (mean cohort age 62 y). (Andrews, Das, Cherbuin, Anstey, & Easteal, 2016) A recent meta-analysis of the effects of *APOE* genotype on cognition in mid life reported no per genotype effect (Lancaster et al., 2017). Only 8 studies with a mean participant age of less than 50 y were included.

Several studies have reported that subjective memory complaints in ε4 carriers aged over 60 are associated with greater decline in episodic memory (Dang, 2017, Dik et al., 2001, Samieri et al., 2014). Caselli et al. found an increased rate of subjective memory complaints in ε4 carriers in their 60 s and older (Caselli et al., 2014). Very recent work from the Australian Womens Healthy Ageing project (age of participants mid 64–77) found that whilst ε4 carriers were not more likely to report subjective memory complaints in response to a single question those that did had more decline in their episodic memory at 2 year follow up (Dang, 2017). Several of these individuals subsequently developed mild cognitive impairment. It is important to note though that there is no consistent relationship between subjective memory complaints and objective decline (Lenehan, Klekociuk, & Summers, 2012). In our study it appears that the increase in subjective memory complaints was also seen in the young people, which runs counter to the suggestion that subjective memory complaints are an early marker of cognitive decline in ε4+ individuals.

There are a number of possible biological explanations for the differences in ε2 carriers. The E2 protein is more abundant than the E4, possibly because it is more resistant to degradation (Conejero-Goldberg et al., 2014). It has reduced affinity for LDL receptors and binds more strongly to Aβ than other ApoE isoforms, which may lead to more efficient clearance of Aβ from cerebral blood vessels (Conejero-Goldberg et al., 2014, Suri et al., 2013). Recent work by Minett et al. suggests that the ε2 and ε4 alleles may have opposite effects on microglial activation (Minett et al., 2016). It has also recently been demonstrated that the E2 isoform of ApoE increased synaptic phagocytosis by astrocytes (with possible evidence of fewer senescent synapses (Chung et al., 2016). It has been shown in post-mortem brain studies and in PET studies of older adults that ε4 allele possession is associated with a higher amyloid plaque load (Caselli et al., 2010, Wirth et al., 2014). In one study this effect was seen even in middle-aged individuals (Ghebremedhin et al., 1998). ε4 effects on neurofibrillary tangles are much less consistent (Raber et al., 2004). Cumulatively this cell biological and neuropathological evidence goes some way towards explaining the opposing effects on the risk of AD seen with the ε2 and ε4 genotypes.

Strengths of this study include adequate screening for dementia, examination of a range of cognitive functions and prospective information on possible confounders. Eleven individuals in this study scored between 82 and 88 on the ACE-R and ten of them were young people. The mother who scored in this range had a borderline low IQ. Educational level is known to affect performance on the ACE-R (Amaral-Carvalho and Caramelli, 2012, García-Caballero et al., 2006, Strydom and Hassiotis, 2003). All of these individuals had an MMSE score of 28 (2 young people) or higher. It is extremely unlikely that an individual in their 20 s would suffer with dementia. We can therefore be confident that no individuals with dementia were included in this study. There were more female than male participants, principally due to the study design. It is possible that ε4 effects on cognition might be more pronounced in women, but this benefit of our study design was almost certainly offset by poor recruitment (Ungar et al., 2014).

The main weakness of this study was poor recruitment. Recall by genotype is an efficient study design which maximises statistical power (Ware et al., 2014). One drawback is that if recruitment is a problem then it is an inflexible design. Despite strenuous efforts to improve recruitment we were unable to recruit further participants. Participants in this study were better educated (mothers), more likely to come from a family that owned its own home and had higher IQs (young people) than the rest of the ALSPAC cohort. The phenomenon of differential loss to follow-up in longitudinal cohort studies is well known and it is the most likely explanation for this finding. It does however limit the generalisability of our findings. There was little evidence of a differential loss to follow-up by *APOE* genotype as the genotypes of all remaining available participants were in Hardy Weinberg Equilibrium (p = 0.016).

In conclusion this study used recall by genotype to study individuals with the full range of *APOE* genotypes. We studied younger individuals than most previous studies and used a wider range of cognitive tests. We did not find any effect of ε4 allele possession on objective cognitive test performance in young to middle aged adults, but they scored higher on the cognitive failures questionnaire (although this seemed to be more prominent in the younger age group). Our findings support the existing literature which suggests that objective ε4 effects do not develop until later adult life and may reflect the earlier onset of AD seen in this genotype group. In addition we found evidence to suggest that ε2 carriers (who have a lower risk of AD) may have mildly superior performance in executive functioning, working memory and episodic memory tasks, domains known to be affected early in the AD disease process.
