# Supplementary material for: Possible positive effect of the APOE ε2 allele on cognition in early to mid-adult life
Source: Neurobiol Learn Mem. 2017 Dec;146:37–46. doi: 10.1016/j.nlm.2017.10.008 (PMC5725639; doi:10.1016/j.nlm.2017.10.008)
Supplement: Supplementary data 1 [file mmc1.docx]

Contents

[Comparison with general population and the rest of the ALSPAC cohort 2](#_Toc485735342)

[Reaction Time results using non-transformed variable 6](#_Toc485735343)

[Comparison between the performance of Mothers and Young People on cognitive testing 7](#_Toc485735344)

[Episodic memory tests 7](#_Toc485735345)

[Executive Functioning 10](#_Toc485735346)

[Working Memory 12](#_Toc485735347)

[Visual Motion Task 13](#_Toc485735348)

[Questionnaire Data 14](#_Toc485735349)

##

## Comparison with general population and the rest of the ALSPAC cohort

As shown in table S1 participants in the recall study were more likely to be home owners (or have mothers who were), more likely to be male, more likely to have achieved a higher education qualification (mothers) and had a higher mean full scale IQ aged 16 (young people). This reflects one of the well-known difficulties with longitudinal studies: differential loss to follow-up. Such differential loss has previously been demonstrated in the ALSPAC cohort.(31)

Table S1: Comparison of the participants in the recall study to the rest of the ALSPAC cohort

| Variable | Whole cohort | | Participant in this study | | Statistical Evidence  T-test, Mann-Whitney or Χ^2^ |
| --- | --- | --- | --- | --- | --- |
|  | Mean | SD | Mean | SD |  |
| Last clinic measured total cholesterol (mmol/litre) | 5.075 | 0.846 | 5.018 | 0.830 | p=0.628 |
| Full scale IQ of YP aged 16 | 91.922 | 13.081 | 95.709 | 12.970 | p=0.011 * |
| Crown-Crisp total score 5 yrs after delivery (mother) | 15.670 | 8.954 | 14.132 | 8.334 | p=0.074 |
| Last available diastolic BP (mmHg) | 69.755 | 9.994 | 70.281 | 10.287 | p=0.605 |
| Last available systolic BP (mmHg) | 122.461 | 13.855 | 122.964 | 15.582 | p=0.723 |
| Last available moods & feelings score YPs) | 38.512 | 5.364 | 38.265 | 5.520 | p=0.866 |
| Home ownership (parents)  *Owned/mortgaged*  *Other* | 6855  2233 |  | 98  10 |  | Χ^2^= 14.2040  p = 0.003 * |
| Gender (whole cohort)  *Male*  *Female* | 5138  14,911 |  | 55  59 |  | p=0.002 * (Χ^2^) |
| Ever had a significant head injury  *Yes*  *No* | 1410  8556 |  | 20  97 |  | Χ^2^= 0.8246  p = 0.364 |
| Social Class of mother  *I*  *II*  *III*  *IV/V* | 413  2272  3645  857 |  | 7  38  45  7 |  | Χ^2^=6.4979  p = 0.370 |
| Mothers highest educational qualification  *CSE*  *Vocational*  *O level*  *A level*  *Degree* | 1652  820  3116  1981  1185 |  | 7  7  44  31  20 |  | Χ^2^=14.0373  p= 0.007 * |

**Stroop Test Results**

Table S2: Results from the computerised stroop test. All reaction times given are cmeans. Χ^2^ refers to statistical evidence from the Kruskal Wallis test.

| Variable | ε2+ | | ε33 | | ε4+ | | Statistical evidence (ANOVA or Kruskal Wallis) |
| --- | --- | --- | --- | --- | --- | --- | --- |
|  | Mean | SD | Mean | SD | Mean | SD |  |
| Stroop interference effect (ms) | 91.878 | 95.07 | 124.454 | 94.421 | 109.775 | 84.805 | p=0.324 |
| Stroop facilitation effect (ms) | -28.289 | 62.063 | -0.677 | 57.611 | -10.103 | 59.918 | Χ^2^=0.706, p=0.703 |
| Incongruent error rate | 0.051 | 0.074 | 0.067 | 0.071 | 0.052 | 0.074 | Χ^2^=2.298, p=0.317 |
| Congruent error rate | 0.015 | 0.028 | 0.017 | 0.031 | 0.01 | 0.021 | Χ^2^=1.180, p=0.583 |

The stroop interference effect was calculated by subtracting the control c-mean reaction time from the incongruent c-mean reaction time. The stroop facilitation effect was calculated by subtracting the control c-mean reaction time from the congruent c-mean reaction time. As anticipated there was a clear effect of the incongruent word colours slowing down reaction times. There was no evidence at all of an APOE genotype effect on performance in the stroop test, as shown in Table S2.

**Visual Motion Task**

**Accuracy**

Table S3: Output from the repeated measures ANOVA for d' versus APOE genotype. As can be seen there was no main effect of APOE genotype on accuracy in the visual motion task. The residuals from this ANOVA were normally distributed.

|  | **Partial SS** | **df** | **Probability >F** |
| --- | --- | --- | --- |
| Model | 49.738 | 117 | 0.000 |
| *APOE* genotype | 0.471 | 2 | 0.588 |
| level | 0.268 | 2 | 0.156 |
| Interaction between APOE and level | 0.800 | 4 | 0.027 |

Table S4: Output from the repeated measures ANOVA for d' versus APOE genotype with participants who got the keys the wrong way round excluded. As can be seen the finding of no association was not altered.

|  | **Partial SS** | **df** | **Probability >F** |
| --- | --- | --- | --- |
| Model | 47.115 | 112 | 0.000 |
| *APOE* genotype | 0.409 | 2 | 0.630 |
| level | 0.165 | 2 | 0.296 |
| Interaction between APOE and level | 0.662 | 4 | 0.047 |

## Reaction Time results using non-transformed variable

Table S5: Output from the regression with the non-transformed reaction time variable. It should be noted that the residuals from this regression were not normally distributed and thus the assumptions of linear regression were violated.

| Covariate | Coef. | Std. Err. | P>\|z\| | 95% Conf. Interval |
| --- | --- | --- | --- | --- |
| Level |  |  |  |  |
| 1* | 0 |  |  |  |
| 2 | 26.414 | 33.039 | 0.424 | (-38.341 to 91.169) |
| 3 | 93.181 | 33.039 | 0.005 | (28.426 to 157.936) |
| Apoe234 |  |  |  |  |
| 33* | 0 |  |  |  |
| 2+ | 175.637 | 121.549 | 0.148 | (-62.595 to 413.869) |
| 4+ | 15.222 | 115.027 | 0.895 | (-210.228 to 240.671) |

A sensitivity analysis was performed excluding 5 participants who appeared to have been confused and to have got the response keys the wrong way round (see supplementary information). This did not alter the results for accuracy or for reaction time.

## Comparison between the performance of Mothers and Young People on cognitive testing

### Episodic memory tests





Figure S1: Performance in the Rey Auditory Verbal Learning Test divided into mothers and young people. Standard deviations for each group are shown in the error bars.

In the whole study group it appeared that those with an ε2 allele seemed to perform better in the RAVLT at the short delay recall task, but that there was no difference at the long delay recall time point. As shown in Figure S1 the difference at the short delay time point appears to have been driven by the young people, although there were more ε2 carriers in that age group. Figure S2 appears to suggest a similar phenomenon in the episodic list learning task, but this is very speculative and impossible to prove.





Figure S2: Performance on the episodic list learning task divided into mothers and young people. Standard deviations for each group are shown in the error bars.

### Executive Functioning

In the whole study group there was evidence that the ε32 group performed faster on the trails A&B test. As shown in Figure S3 this seems to have been present in both age groups.

####



Figure S3: Performance on the trails A&B test divided into mothers and young people. Standard deviations for each group are shown in the error bars.

### Working Memory

The 3-back task was chosen to examine whether there appeared to be a difference between YPs and the mothers. This was because of the greater difficulty of this task which resulted in a wider spread of the accuracy results. As can be seen from Figure 4 it appears that the greater accuracy in the ε22 group was seen in both age groups.





Figure S4: Accuracy in the 3-back task for Mothers and YPs. Standard deviations for each group are shown in the error bars.

### Visual Motion Task

In the main study there appeared to be an increased cmean reaction time in the ε32 group, which was most apparent in the target absent and medium speed condition. Figure S5 shows the same pattern as the main study. The only apparent difference is that the YPs had generally slower reaction times in the slow condition.





Figure S5: Cmean reaction times for the target absent condition divided into Mothers and YPs.

### Questionnaire Data

As shown in Figure S6 it appears that ε4 allele possession had a greater effect on scores on the depression, anxiety and stress scale (DASS) in mothers. This is intriguing as it measures some of the same constructs as the Crown-Crisp scale, which was only completed by the mothers. There was no evidence of a per genotype difference in the mothers on either the Crown-Crisp scale or the Edinburgh post-natal depression scale (see Table 1). In the whole study group there was evidence of a higher total score in the cognitive failures questionnaire (CFQ) in the ε34 group. Surprisingly this difference would seem to be more marked in the younger people (see Figure S7).





Figure S6: Total score on the depression, anxiety and stress scale split into mothers only or young people only.





Figure S7: Total score on the cognitive failures questionnaire in young people and mothers.
